# Disability, spirituality and belonging in Africa: An intersectional and decolonial perspective

**DOI:** 10.4102/ajod.v15i0.1796

**Published:** 2026-06-18

**Authors:** Chioma Ohajunwa, Adele Ebrahim, Nafisa Mayat

**Affiliations:** 1 Africa Centre for Inclusive Health Management, Faculty of Economic and Management Sciences, Stellenbosch University, Cape Town, South Africa; 2 Division of Occupational Therapy, Department of Health and Rehabilitation Sciences, Faculty of Health Sciences, University of Cape Town, Cape Town, South Africa; 3 Division for Disability Studies, Department of Health and Rehabilitation Sciences, Faculty of Health Sciences, University of Cape Town, Cape Town, South Africa

The African continent is characterised by profound religious and spiritual diversity – Christianity, Islam, indigenous African Traditional Religions (ATRs) to name a few and a variety of syncretic spiritualities that all shape how individuals and communities make sense of life, suffering, embodiment and difference. For many African communities, spirituality and religion are not peripheral; they are central to identity, personhood and belonging. Yet, disability in these religious contexts is too often framed through stigmatising or pathologising perspectives, shaped by complex intersections of colonial legacies and local understandings.

This special issue of the *African Journal of Disabilities* seeks to foreground the intersections of disability, spirituality, religion and belonging, with the aim of reframing dominant narratives and bringing forward voices that have been historically marginalised.

In numerous African contexts, disability is constructed as a spiritual identity. Local beliefs frequently link disability with ancestral displeasure, witchcraft or divine punishment (Ingstad & Whyte 2007). These interpretations are deeply embedded in traditional cosmologies but have also been influenced – and, in some cases, exacerbated – by the imposition of colonial religious ideologies (Grech 2015; Ned 2022). Euro-Christian missionary movements often pathologised both indigenous spiritual practices and disability itself, reinforcing theological models that equated disability with sin, deviation or moral failing (Behrens 2013; Ndlovu-Gatsheni 2018).

This special issue calls for a critical re-examination of such inherited ideas through a decolonial lens – one that challenges Eurocentric constructions of disability and recognises the epistemic value of African spiritual knowledge systems (Chilisa 2012). Decolonisation, in this context, is not merely about reclaiming tradition but about interrogating persistent hierarchies of ability, normalcy and personhood in postcolonial religious institutions and development frameworks.

Drawing on the framework of intersectionality (Crenshaw 1991), the contributions in this issue illuminate how experiences of disability are shaped by overlapping and intersecting identities – gender, class, age, geography and religious affiliation. For example, women with disabilities often navigate multiple layers of marginalisation within patriarchal religious contexts, where both their physical and spiritual capacities may be scrutinised or undervalued (Erevelles & Minear 2010). Similarly, youth with disabilities may find themselves excluded from initiation rituals or communal worship, undermining their sense of social and spiritual belonging.

For many persons with disabilities, spirituality provides more than doctrine – it serves as a source of identity, healing and relational belonging. The contributors to this issue demonstrate that African interpretations of spirituality and community, although sometimes fluid and very much metaphysical, offer resources for constructing more inclusive theologies and spiritual practices (Dwadwa-Henda, Mji & Ohajunwa 2025).

The goal, then, is not to romanticise either traditional or religious frameworks but to engage them critically and contextually, seeking emancipatory possibilities while resisting interpretations that diminish the value of lives with disability. Spirituality can function as a decolonial praxis, a means by which persons with disabilities reclaim space, meaning and agency in the face of exclusionary theologies and policy frameworks. The decolonial lens is one framework through which persons without disabilities can interrogate their own narratives on disability and how these narratives enforce exclusionary practices within the spiritual and faith-based spaces.

True transformation requires more than superficial inclusion; it demands full participation, theological reform and leadership by people with disabilities themselves. Decolonisation and intersectionality together call not simply for a seat at the table, but for a re-envisioning of the table itself – one where African spiritual traditions, disabled voices and communal ethics co-create justice and flourishing.

This special issue is not simply an academic exercise. Rather, it is an invitation to scholars, faith leaders, policymakers and practitioners to critically engage with the colonial and ableist legacies embedded in religious practices and academic paradigms. We thank our contributors for their intellectual rigour and lived wisdom. The journey towards inclusion, belonging and justice for persons with disabilities in African spiritual contexts is far from over – and must be led by those whose lives and voices have too often been marginalised.

Submissions for the special issue covered a range of narratives and experiences of disability and inclusion and how these are influenced by the intersecting identities of persons with disabilities within various contexts. The articles offer an alternative lens of spiritual resiliency that has the capacity to facilitate an inclusion for persons with disabilities, which supports a sense of belonging. The Judeo-Christian Church, Islamic religious belief systems, African Indigenous belief systems and the spiritually informed processes that influence inclusion within these religious spaces for persons with disabilities are critiqued and discussed. The role of Africa’s colonial history and its influence on religious expression and positioning of persons with disabilities is presented as an intersecting influential factor. However, the current potential use of digital platforms to facilitate spiritual spaces of inclusion is equally highlighted.

## Disability, spirituality and the politics of belonging in postcolonial Zimbabwe

In this article, authors explore the intersection of disability, spirituality, belonging and citizenship in Zimbabwe and how the understanding ascribed to these concepts creates a complex cultural, socio-political contexts that impact on the inclusion and exclusion. Authors use Marco Antonsich’s conceptualisation of place-belongingness and the politics of belonging to explore within the literature how persons with disabilities make emotional connections to places and spaces, even when experiencing exclusion from those spaces. Authors present that disability in Zimbabwe must be understood from history and context, citing colonial history and current contextual ambiguity and overlapping identities inherited from this history. They argue for a more spiritually aligned approach (grassroots and Ubuntu ethics) to inform disability inclusion in Zimbabwe.

## Baptist church and empowerment of women and girls with disabilities in Mhondoro-Ngezi

In this article, authors bring up the intersectional gendered dynamics within the Judeo-Christian and Shona indigenous religious spaces. Authors argue that women and girls with disabilities experience more stigma and marginalisation based on their gender and disability. The article identifies spirituality as a capital that can be used to foster greater social and economic inclusion in Zimbabwe, outlining how the Ngezi Baptist Church is using spiritual narratives to disrupt these narratives and lens of oppression on women and girls with disabilities. The article utilised a feminist disability theology approach and focus groups and in-depth interviews to explore this notion of a spiritually mediated inclusion within the Baptist church in Ngezi Zimbabwe. The Church encourages a reimagining of God as contextual, a multi-dimensional presentation of God as reflecting all forms of human variation, which includes gender, able-bodied or differently abled. This thinking informs a validity of the self that contributes to a sense of belonging.

## The potential and challenges of an online Bible study group for people with disabilities

This study explores the use of digital spaces within churches to facilitate the inclusion of persons with disabilities. The authors employ a phenomenological research approach, with semi-structured interviews to investigate the benefits and challenges of using the online space as a facilitator of religious inclusion for persons with disabilities. They reiterate the importance of religious inclusion of persons with disabilities, as this supports the human needs for purpose, love and belonging. The achievement of these human needs through spiritual inclusion further promotes coping, social inclusion and better quality of life for persons with disabilities. The article highlights the challenges of transportation and physical accessibility faced by persons with disabilities in attending the physical church. However, access to the church’s spiritual community was perceived to be very beneficial in creating a sense of belonging and community. Participants refer to a sense of emotional and spiritual guidance and support, which creates a sense of safety for them. The study reiterates the use of technology and digital platforms within churches to include people with disabilities, while also pushing for attitudinal changes within religious organisations.

## Pesantren and inclusion: Bridging religion and disability in Islamic education in Indonesia

In this study, authors implement a literature review method, drawing on historical analyses, curriculum evaluations and policy assessments related to inclusive education in pesantren, a fundamental pillar of Islamic education in Indonesia. The authors discuss the evolving nature of pesantren and how it is promoting inclusive education, based on Islamic religious principles. Although pesantren has not historically been inclusive, it is currently beginning to play a crucial role in fostering tolerance and a broader understanding of diversity at the intersection of the intersection of religion, education and social inclusion. The authors argue that pesantren is an example of the capacity of traditional religious institutions to evolve to meet contemporary education demands for persons with disabilities. This has implications for policy, curriculum, religion and inclusion.

## Power dynamics in African spirituality and disability: The South African context

Utilising a qualitative research approach and a comprehensive literature review, the author explores the intersection of disability, spirituality and power within African spirituality in South Africa. This is done through the theoretical lenses of Foucault’s Power and Knowledge theory, Intersectionality and Inclusion and exclusion theory. The article explores the power dynamics that permeate African spiritual beliefs and practices within the South African context and the complex narratives surrounding this belief system, which influence the lived experiences of persons with disabilities within this context. The author highlights the capacity of African spirituality and belief systems to both empower and marginalise persons with disabilities. The study presents the dual role of spirituality in the lives of persons with disabilities, situating spirituality as a source of strength and resilience, but has equally been weaponised against persons with disabilities, by some religious entities and communities. The article argues that within African spirituality, rituals and observances are performed for healing and restoration, while certain impairments are viewed through a lens of spiritual deficiency and stigmatised. The article advocates for an intentional strategy that emphasise the positive use of spiritual principles to foster purpose, inclusion and community.

Certain factors about the relationship between disability and spirituality emerge within this special issue, a major factor being that spirituality supports coping and resilience for persons with disabilities. Although the discussion of spirituality is quite complex within the African context because of the history of colonialism among others, spirituality informs the lens persons with disabilities view the world around them and equally view themselves and their experiences with Mugeere et al. (2020). Spirituality also informs a sense of agency and positioning of persons with disabilities (Lorenzo & Duncan 2020), hence, a very strong tool for supporting the lived experiences of persons with disabilities.

## Conclusion

This special issue, as presented herein, has emphasised the powerful influence of spirituality on the lived experiences of persons with disabilities. Spirituality supports a sense of belonging, safety, community and emotional resilience and coping for persons with disabilities. However, on the other hand, religious institutions have been guilty of stigmatising and marginalising persons with disabilities. The critical importance of ensuring attitudinal and physical accessibility cannot be understated, and the digital platform has been cited as one space that could support access to spiritual spaces and communities. The need to recognise the legacy of coloniality and its positioning of persons with disabilities on a deficit, including the dual roles played by African spirituality and the faith communities in general, was highlighted. One major outcome of this special issue is the need for intentional, spiritual practices that are inclusive of persons with disabilities, recognising the capacity of spirituality to support inclusion, a sense of belonging and active citizenship.
